# Mistaking the Map for the Territory: What Society Does With Medicine: Comment on "Medicalisation and Overdiagnosis: What Society Does to Medicine"

**DOI:** 10.15171/ijhpm.2017.20

**Published:** 2017-02-19

**Authors:** Alistair Wardrope

**Affiliations:** University of Sheffield, Sheffield, UK.

**Keywords:** Medicalisation, Philosophy of Science, Biomedical Model

## Abstract

Van Dijk et al describe how society’s influence on medicine drives both medicalisation and overdiagnosis, and allege that a major political and ethical concern regarding our increasingly interpreting the world through a biomedical lens is that it serves to individualise and depoliticize social problems. I argue that for medicalisation to serve this purpose, it would have to exclude the possibility of also considering problems in other (social or political) terms; but to think that medical descriptions of the world seek to or are able to do this is to misunderstand the purpose and function of model construction in science in general, and medicine in particular. So, if medicalisation is nonetheless used for the depoliticization described by many critics, we must ask what society does with medicine to give it this exclusive authority. I propose that the problem arises from a tendency to mistake the map for the territory, and think a tool to understand certain aspects of the world gives us the complete picture. To resist this process, I suggest health workers should be more open about the purpose and limitations of medicalisation, and the value of alternative descriptions of different aspects of human experience.

## Introduction

According to van Dijk et al, “what society does to medicine” is engage in a reciprocal relationship with healthcare and research institutions that drives both (over)medicalization and overdiagnosis.^1^ They emphasise one key function of this process; the individualization of social or political problems. But for medicalization or overdiagnosis to fulfil this role requires medicalized interpretations of phenomena to exclude other understandings; yet the way health workers and researchers employ biomedical knowledge is not exclusive in this fashion. Thus we must ask also “what society does *with* medicine” – exploring how the conceptual resources of medicine are appropriated and transformed to serve certain social functions.

I suggest that one important factor here is the inappropriate reification of biomedical models of human phenomena – the assumption that the medical descriptions of human experience “carve nature at the joints” and incorporate everything that can meaningfully be said about these experiences. This reification, I argue, mistakes the map for the territory – misunderstanding fundamentally the nature and purpose of model construction in healthcare (and science generally).

## The Map and the Territory: Model Construction in Science and Medicine

Usually (though not invariably), to medicalize is to *pathologise* – to label some phenomenon a disorder. Overdiagnosis, too, concerns, what is called ‘disorder’ – since with many conditions (eg, chronic kidney disease or diabetes, or van Dijk et al’s preferred cases of dementia and learning disability),the definition of disorder involves drawing a sharp line in an otherwise fuzzy spectrum.^2^ But, as van Dijk et al correctly highlight, there is no uncontroversial definition of what it is for some condition to be a disorder or disease. Thus we must consider what medicalized descriptions of phenomena – the creation and application of a ‘biomedical model’ – seeks to do. And to achieve this requires understanding the meaning of *model construction* in the biological sciences.

A naive realism would hold that scientific descriptions of phenomena perfectly capture an underlying reality, and its categories – to borrow the Platonic metaphor – “carve nature at its joints.” But to insist that our scientific models adhere to this demanding standard is both to fail to describe actually existing scientific practice and to impose unwarranted limitations on the kinds of work scientific descriptions can perform. Throughout the natural sciences, useful and widely-applied theories are full of entities and pictures that are *fictional constructs*. Some of them invoke concepts that do not demonstrably correspond to any particular entity in their domain of interest (eg, some thermodynamic potentials),while others make deliberate distortions to properties of the object of interest (modelling a pendant as an ideal pendulum, or the world as mapped in the Mercator projection), or even (as with Bohr’s and Rutherford’s models of the atom) directly contradict the theories in which they are grounded.^3^ The way components of models are delineated and their interactions described has as much to do with the purposes for which a given phenomenon is being modelled, as with the nature of the phenomenon itself. These models have nonetheless served as central components of the image of the world presented by their respective sciences, because they *usefully serve the purposes of scientific inquiry* in their respective domains. Scientific modelling is a *perspectival* process^4,5^; it presupposes a vantage point from which we seek to situate phenomena in our understanding of the world, and depends upon the uses to which we seek to put that understanding.

There is good reason to think this is true also of our models of physiological and pathophysiological processes. At times, the same physiological systems are represented in contradictory ways for different purposes: in understanding blood pressure autoregulation – and the pathological states of hypo- and hypertension – blood flow is commonly treated as the laminar flow of an incompressible fluid through cylindrical pipes; but for other purposes such as modelling oedema, capillary permeability or fluid turbulence become more prominent. At other times systems are described at different levels in ways such that higher-level explanations can be realized by multiple lower-level systems and are not reducible to any number of these; the functional architecture of the brain as usually invoked in describing symptoms of stroke is neither reducible to nor uniquely explicable in terms of neuronal-level networks.^6^ In other cases, medicine has moved away from a taxonomy grounded in tangible biological differences in favour of delineating diseases with reference to results of standard investigations – eg, the rejection of the old ‘transmural’/’subendocardial’ classification of myocardial infarction (MI) for ST-elevation and non-ST-elevation MI, or the greater preference for the single diagnosis ‘chronic obstructive pulmonary disease’ over ‘chronic bronchitis’ and ‘emphysema.’

What does this tell us about the ‘biomedical image’ of the world? That it is – and only intends to be – a partial, perspectival, and pragmatic attempt to model things going on in the world, in ways that best serve the epistemic and practical objectives of physiological and medical practice – chief among these being prediction (of how intervening in a physiological system will alter its behavior, or clinically how a given condition may develop) and technological intervention (understanding what interventions might help people become healthier). Biomedical models of human experience are not complete descriptions of the territory – they are maps to help guide us through it.

## Mistaking the Map for the Territory

This view of the biomedical image is at odds with one of the most pervasive critiques of both medicalization and overdiagnosis – the claim, endemic throughout the medicalization literature, that medicalization serves to individualise social problems, to “locate the sources and solution of these problems increasingly on the individual level.”^1^ For this to be a strict logical consequence of medicalisation alone would require that the biomedical description of a phenomenon is an exclusive one – taken to describe the ‘true’ nature of the thing, without remainder.^7^ If the above picture of biomedical models is correct, though, this is not the case.

Even if not logically entailed by medicalisation, the *nonlogical* implications of the way medical institutions speak about conditions might serve this purpose. If in practice health workers overwhelmingly focus on the individual and biological (despite ostensibly acknowledge the significance of the social or political) then this might form an ‘ideology’^8^ that, combined with the socially privileged position of medical institutions, serves to exclude those other dimensions in practice from collective understanding^ [1] ^.^3^ This however cannot be the whole story, since – as van Dijk et al themselves observe – health workers generally do *not* treat medicalization as excluding social understandings of a phenomenon. Indeed, they are often amongst the most vocal advocates for political action on supposedly medicalized issues.^1^

If the mere fact of medicalization does not entail this individualization, we need to ask *how* medicalisation might be further exploited to produce this result. In other words, we must take up Ann Garry’s challenge to “disentangle a number of other social factors that play into the negative constructions that surround or at least accompany medicalisation: commercialization, risk management, …”^10^ – to determine what society does *with* medicine and the biomedical image of the world in order to achieve this result. To make this leap from medicalisation to biomedical reductionism requires reading a pragmatic model as the complete picture – mistaking the map for the territory.

This mistake is practically demonstrated throughout the uses to which medical descriptions are put in different settings. As George Szmukler observes, diagnoses are hugely “overworked tools.”^11^ They are used, *inter alia*, to: determine eligibility to certain forms of state benefit; to evaluate insurance policies and claims; and to determine degree of criminal responsibility in the courts. If these diagnoses did represent the ‘territory’ – they completely and accurately described the lives and experiences of the people bearing them – then this might be legitimate without further argument. But if in fact the biomedical image is tailored to serving entirely different purposes – those of medical research and clinical practice – it gives no reason to believe it can usefully serve these additional functions, any more than a screwdriver can be used as a saw^ [2] ^.

If, then, (as van Dijk et al suggest) diagnoses of ‘mild cognitive impairment’ or ‘mild learning disability’ drive institutionalization of people struggling to cope with the complexities of contemporary society –the “medicalized answer of a society that ultimately values economic efficiency over inclusiveness”^1^ – the source of this problem lies not with the *medicalisation* itself (since health workers explicitly acknowledge these diagnoses are culture-bound, defined in terms of function within a society and – in the case of LD – relativized to community statistical norms). The problem, rather, is with what society does *with* those descriptions – treating them as excluding other understandings and so licensing an exclusively medical engagement with the relevant problems.

The same mechanic is played out repeatedly in many other controversial medicalisation cases. Given a basic assumption of materialism, it should not be controversial to assert the importance of physiological processes to explain why some people misuse and become addicted to alcohol or other substances, or that intervention at a physiological or psychological level might help individuals struggling with addiction; the problem arises only when describing addiction as a physiological phenomenon is taken to entail that it is *not* a social phenomenon.^12^ In a rather different setting, patients with medically unexplained symptoms appear to seek from medical descriptions of their experience, not accurate predictions or successful treatment, but rather a legitimization of their suffering and removal of blame.^13^ Here medicalisation functions not to guide treatment but to “give something [social] acceptance and status.”^14^ There are useful medical descriptions of human pain that in many cases are of great benefit to people – but when these descriptions are taken to be the only legitimate ones, and medicine granted a monopoly on human suffering, then it is inevitable that people will seek to interpret their distress through a medicalized lens.

## Conclusion

If what society does *with* medicine is to reify the biomedical image of the world in a way that inhibits political responses to social problems, how are those responsible for shaping the biomedical image to respond? Van Dijk et al describe a vicious cycle involving medical practitioners and institutions and the societies in which they are situated that drives increasing medicalisation and overdiagnosis; but recognition that this cycle depends upon mistaking the medical map for the human territory affords an opportunity to break it. Van Dijk et al suggest that an increasing medicalisation of human experience at the macro-level makes individual people more likely to interpret their own experiences in medicalized terms, and to seek medical remedies for it; the resulting expansion in rates of diagnosis raises the prominence of medical descriptions of the relevant dimensions of people’s lives, encouraging further medicalisation. This cycle is driven by the move from medicalisation to the interpretation of human experience overwhelmingly in medical terms; but if the above argument is correct, the latter is not an inevitable consequence of the former. To prevent this inference requires an increased individual and societal awareness of how the biomedical image of the world only provides a limited perspective upon it – one enormously valuable for serving certain purposes, but utterly unsuited to others. With this established, the medical lens becomes one amongst a range of complementary perspectives through which to view the world.

A partial response to the harmful consequences of what society may do with medicine, then, involves humility on the part of medical professionals and institutions – a frank admission of the limitations of the biomedical image of the world, and an embrace of other images that are better able to serve other purposes, at least as important to individual and social flourishing.

## Ethical issues

Not applicable.

## Competing interests

Author declares that he has no competing interests.

## Author’s contribution

AW is the single author of the paper.

## Endnotes


[1] I am grateful to an anonymous reviewer for highlighting the need to acknowledge the operations of epistemic and social privilege in this context. Unfortunately a more detailed exploration of these issues lies beyond the scope of this paper, but is a subject of much recent work on the subject of epistemic injustice and illness.^9^

[2] An anonymous reviewer highlighted that these uses of diagnoses do not necessarily require the realist interpretation of medicalised descriptions of these phenomena; one may simply declare by fiat that eg, ‘if you have diagnosis x, you are entitled to benefit y.’ This is, of course, correct; my point concerns rather the justification of such policies. If saying that someone has diagnosis x is to say that they simply are a certain way, then one might be able to infer eg, what responsibilities the state has toward them, or what actions they should or should not be held criminally responsible for. But if their having diagnosis x is only to say that we can expect them to display certain biological (ir)regularities, and that certain treatments may have particular benefits for them, then it is illegitimate to infer without further argument that this should entail anything about their standing with regard to these separate social or political questions.

